# Parenting, privilege, and pandemic: From surviving to thriving as a mother in the academy

**DOI:** 10.1177/1473325020973328

**Published:** 2021-03

**Authors:** Haley H Beech, Amber Sutton, Leah Cheatham

**Affiliations:** School of Social Work, University of Alabama, Tuscaloosa, USA

**Keywords:** Critical reflection, mothering, collaboration

## Abstract

As women who have dual roles as parents and academicians, COVID-19 has presented new challenges and opportunities that have impacted our personal and professional lives. This essay provides insight into unique obstacles from the perspective of mothers, researchers, and social workers and challenges the traditional models of work/life balance as professionals in academics. This reflexive essay provides the narratives and experiences of one assistant professor and two doctoral students who are learning to navigate motherhood and professional responsibilities amidst a global pandemic. The prologue presents a perspective from a current assistant professor and her lived experiences followed by the reflections of two doctoral students on how to navigate the academy as mothers and as women. In addition to our personal stories and narratives, we hope to challenge, inspire, and reimagine how our dual roles can be viewed as an asset, rather than a weakness and encourage others in the academy to rise and support women.

## Prologue

 Reflecting upon the COVID-19 pandemic and how it has affected my work, particularly as a tenure-track assistant professor who is also a mother of two young children, I cannot help but be grateful for some of the social shifts occurring within the academy, if not the working world. I hesitate to admit this, given the heartbreaking loss of life experienced across the globe, as well as the devastating economic consequences associated with this pandemic. However, as a strengths-based thinker, I recognize that some good may have found its way in.

### Pre-COVID-19 perspective

Beginning this academic journey, I had few mentors who were also mothers—one, to be precise. At certain junctures of my doctoral program, I wondered if academic life and motherhood were realistically compatible. Once I became a mother halfway through my PhD studies, I struggled to minimize the influence of my newly acquired role; scheduling calls around feeding times or naps and avoiding casual references to my little ones during meetings. Toward the end of this well-coordinated act, I secured a tenure-track position within a predominantly female unit, where many tenured/tenure-track faculty were mothers. Yet, despite this new environment, old habits lingered along with self-doubts.

My transition from student to faculty was tumultuous. My oldest child, diagnosed with Leukemia, needed me more than ever before. While attempting to balance these roles—junior faculty, mother, and caretaker—I received ample reassurances from my new colleagues. Nevertheless, I recall feeling acute guilt when I was torn away from my role as professor to carry out my role as mother and caretaker. I remember hesitating to disclose when I was working from my son’s hospital room, for fear colleagues would think less of me as a professional. This fight to ensure hard lines were enforced between my multiple roles was exhausting.

### Post-COVID-19 progress

The global pandemic has offered me, and hopefully many others, an entirely novel perspective and with it the permission to leave behind many of these tendencies. By presenting a crisis to all, COVID-19 has shifted the ways in which we work and, in doing so, has reinforced the bonds of our humanity. Professional has merged with personal, as we join meetings from kitchens, porches, and home offices. Too many of us have experienced “guest appearances” by family on Zoom;the small child bust-ins that once gave me panic attacks are now par for the course. We’re learning to see each other as multifaceted individuals, rather than unidimensional professionals. And for many of us who once felt the need to downplay our commitment to family within a profession that prizes intense productivity, we now take comfort in knowing that the world, in this long pandemic-induced pause, has rightly remembered that these close relationships define our identities and are central to our daily existence.

### A vision for the future

Along with hardship and sorrow for countless individuals, this pandemic has brought moments of reflection and recognition. From this new vantage as a junior faculty member I have the privilege of witnessing the vision and drive of graduate students who are also mothers. While they, too, are experiencing challenges along their academic journey (many more daunting than my own), I see that this next generation of mother-academics is unwilling to censor their identities as I too recently felt necessary. Amidst this pandemic pause, they are envisioning a more diverse, inclusive, and equitable academy: one that recognizes mothers’ abilities to balance multiple demands as a strength, particularly well-suited to the academy where, in furtherance of research, teaching, and service, we naturally find ourselves balancing numerous roles. Instead of surviving in the academy despite being mothers, these students are thriving in the academy because they are mothers.

As many of us return to campus and struggle to create a “new normal”, we should also reflect be careful not to forget the lessens brought by COVID-19, which offer valuable guidance as to how we can better support the valuable contributions of mothers within the academy. The actionable suggestions articulated in the following essay are well-aligned with our professional ideals as social workers, valuing diverse experiences, and affording equitable opportunities to all. If we can hold tight to lessons of shared humanity through this global crisis, we may be fortunate enough to find a more inclusive and supportive academy on the other side.

## Introduction

**Figure 1.**
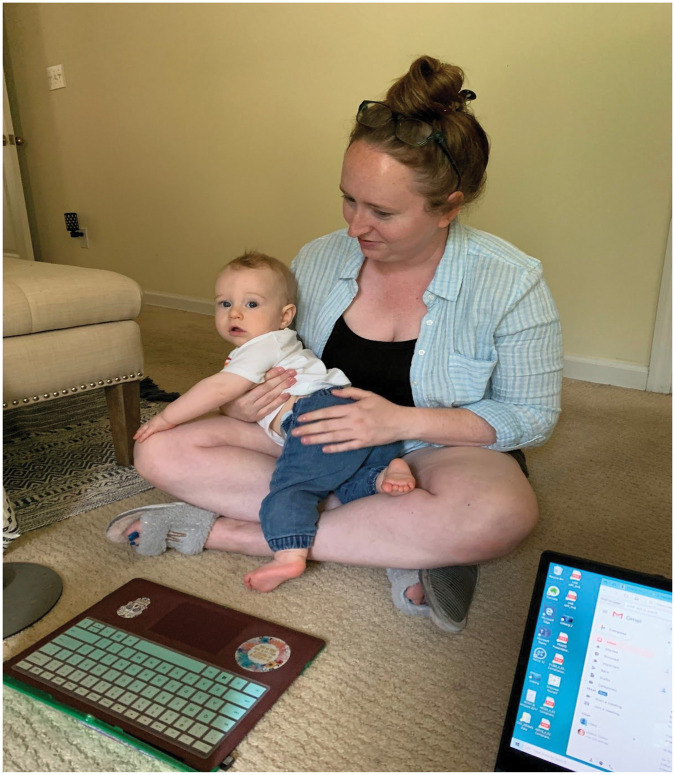
Amber Sutton works with Haley Beech while parenting her son from home.

As COVID-19 has halted and disrupted the lives of many, mothers in the academy have experienced unique challenges that directly impact their personal and professional lives (Figure 1). Finding balance between parenting and working from home has created new opportunities, but also many barriers including remote work, professional calls in personal settings, navigating shared spaces, and the isolation of working from home. As social workers, PhD students, researchers, and mothers, we aim to share our lived experiences of navigating the tumultuous waters of academic life from our homes in the era of COVID-19. We acknowledge and freely share that our personal experiences are situated within our own privilege and intersecting identities as White, cisgender, heterosexual mothers of young children whose research focuses on the intersect of maternal health and intimate partner violence. Many of our lived experiences are shared experiences with those we work with daily in the community and in the ivory tower. We hope to share our perspective on these unprecedented times and aim to bring hope and inspiration to other mothers.

### Strength-based perspectives in a pandemic

We would like to begin by sharing several positive outcomes from this pandemic and how this global crisis has shaped and reshaped our views of academic life as we once knew it. From this new paradigm, we have found more liberty and freedom to explore creativity in our academic endeavors as many of our community-based projects have been placed on hold and refocused our energies to explore alternative outlets for shared research knowledge, not only through peer-reviewed publications. While we are still moving academic publications forward, we have focused our energies towards call to actions, community partner co-publishing, and reflexivity. In addition, this season of life has also helped us find creative outlets for sharing knowledge by embracing non-academic spaces including blogs, podcasts, Op-Eds, public forums, and local media outlets. COVID-19 is teaching us that the gap between academic knowledge and local knowledge should be closing in, not widening. It is more imperative than ever to value the lived experiences of community members and to provide space for alternative voices in the academy. These approaches have taught us to break out of the traditional PhD/academy model that is hierarchical, linear, and often fraught with patriarchal values. Additionally, COVID-19 has exposed cracks in the system that highlight chronic, systemic injustices in the academy that prioritize and favor privileged identities, not excluding ourselves: highly educated, White women. The time has come to create equitable opportunities to rebalance privileged voices and identities by welcoming other perspectives and lived experiences outside the clearly defined lines of academic walls.

### Obstacles in a pandemic

We have also faced many barriers that have made it difficult to find work/life balance. Our home spaces have now become our workspaces by which we have to navigate constant interruptions including the need to be available for our partners and children, sometimes at the cost of our own productivity. There are peculiar feats of working from home, particularly when you are in the midst of preparing for a Zoom call, changing a diaper, and hustling to gather yourself with five minutes to spare, all the while realizing that you forgot to wipe baby food and spit up off your shirt. Living through a pandemic has awakened us to reckon with and accept our reality of having dual roles and of our ability through vulnerability to close the dichotomous gap between professional and personal. We continue to introspectively challenge our positionality, relatability, and allow our lived experiences as social workers, researchers, and mothers to inform and transform our work with other mothers. We embrace not having a compartmentalized, idealistic view of ourselves as professionals and mothers through a daily concoction of empathy, grace, and self-compassion. It is messy and complex and COVID-19 has given women who are mothers permission to blur the lines between these identities uncovering that our personal has become our professional and vice-versa. With the caveat, based on your identity, these complexities will look different for different women. For us, it looks like navigating these fluid identities and becoming aware of which spaces are safe or unsafe. As we have learned, safety is never guaranteed and is often a privilege, not a human right.

### The professional is personal

Through these unprecedented times brought on by a worldwide pandemic, we are learning what it looks like to redefine our professional and personal identity as social workers, academic professionals, and mothers. We should not and do not have to choose between having a career and being a mom. We can have both even within the academy as long as we take ownership of our boundaries and redefine what balance means to us. Our vulnerability is our victory. We have relearned what it means to find comfortability and empowerment within the struggle and to create common ground for our fellow mothers. We must redefine the new normal by allowing our multiple identities to teach and inform us in all aspects of our lives. We also aim to support other mothers in these dual roles by creating safer spaces that allow for success at work and at home. The time has come to stop forcing women to choose which identity will take priority and supporting mothers to embrace all of who they are, as a strength, not a weakness. All women deserve respect, and as academicians and mothers, we will no longer stand by and be treated as anything less than the brilliant, multitasking, badass mothers we are.

### A new academic paradigm

The new normal for mothers in the academy can no longer overlook barriers to our success such as childcare, lack of financial support, particularly as PhD students, and accommodations for children in the workplace such as childcare, nursing rooms, changing stations, play areas, and appropriate supplies. If addressed, these structural shifts can send an important message: that your identity as a mother matters and that you are supported in your journey to integrate these identities throughout your career. Being a mother in the academy should not be viewed as a setback, barrier, or be perceived as an indication of low professional commitment. Rather, motherhood should be viewed as a strength, gift, and suggestive of an ability to find balance. This strengths-based perspective must be the new normal moving forward. As social workers, we believe there is a higher standard and calling to our profession and schools of social work to be at the forefront of these shifts and begin the movement of welcoming mothers and children into academic spaces. We must support mothers and redefine what it looks like to be successful in academic circles.

### The bond of motherhood

Personally experiencing motherhood has allowed us to be more compassionate with the women we work with and with ourselves. It has also allowed for an inner patience that goes beyond an intellectual understanding and can only be acquired through a visceral bond between mothers. In our work, specifically with mothers who have experienced violence, a new empathy and appreciation has emerged for the struggle of often having to choose between safety of themselves and the safety of their children. Motherhood has opened our eyes to how women and mothers in general are not prioritized in society, thus leading to the diminished experience of others, including their children. We must support and prioritize mothers by choosing to see women as whole beings, rather than a sliver of their existence. Women are not a monolithic group and we must renounce society’s rigid constructs that restrict autonomy and treat mothers as commodities of their bodies. Celebrating our intersecting identities leads to accepting and loving women. We are called to pay tribute to the sacred power of mothers as nurturers, healers, and the heartbeat of communities. We must continue to find ways to celebrate motherhood and amplify mothers’ voices rather than devaluing their gifts. Mothers, we see you and we honor you.

## Conclusion

Throughout this pandemic, we learned the only thing that is ever certain is uncertainty and that now more than ever, there is a need for a space that invites empathy and diminishes isolation. For mothers there is a natural built-in support system when you connect to other moms. There is, oftentimes, an unspoken and unique pact that bonds us; A “me too,” if you will, that signifies a mutual understanding and relatability between people; people that may otherwise never cross paths and that have distinctive intersecting identities. These shared spaces create authenticity and give women the permission to just show up as we are, to embrace the messiness of the world we occupy, and to reject the notion of categorizing our lives into neat and tidy packages. Maybe what COVID-19 revealed most of all is that, as doctoral students and mothers, we had everything we needed all along. Motherhood has prepared us to excel as professionals. Everything changed and yet, nothing changed at all. Being a student, professional, and mother does not change in a pandemic, it only magnifies what we and thousands of other women do daily. It gives us pause to slow down, catch our breath, and celebrate who we are and the unique gifts that we bring. The answers to our lives are not necessarily meant to be found outside of ourselves. The answers we are looking for can often be found in our own inner worlds. Our experiences can serve as an example and a reminder that mothers do have a place in the academy and that strength can look different for each individual. We belong.

